# Satellite Symposium on Challenges to Enteric Vaccines: Salvador de Bahia, Brazil, 12 June 2005

**Published:** 2007-03

**Authors:** A.D. Steele, M.P. Kieny

**Affiliations:** Initiative for Vaccine Research, World Health Organization, Avenue Appia 20, CH-1211 Geneva 27, Switzerland

## Background and rationale

In an effort to promote vaccine research and development of enteric vaccines, including cholera, typhoid fever, and rotavirus vaccines and to address the challenges for their future implementation, the WHO Initiative for Vaccine Research hosted a one-day Satellite Symposium dedicated to addressing the opportunities to use the licensed vaccines against cholera and typhoid fever and to consider the challenges which remain for introduction of rotavirus vaccine, including clinical trials in developing-country settings. The Bill and Melinda Gates Foundation graciously provided US$ 50,000 to organize the Satellite Symposium.

The Symposium was followed by a three-day Global Vaccine Research Forum, which also included a session on the other two important enteric vaccines against enterotoxigenic *Escherichia coli* and *Shigella*. The Forum brought together a world-wide selection of top researchers and scientists and served as a forum for the partners of GAVI (the Global Alliance for Vaccines and Immunization) to discuss vaccine research and development issues and to update research agenda. Moreover, both the meetings provided an opportunity for discussion of broader issues of vaccine policy and implementation.

## Goals, objectives, and activities

Although diarrhoeal mortality, globally, is observed to be on the decrease, it is well-recognized that the vast bulk of morbidity and mortality of the estimated annual 1.9 million deaths is borne by infants and young children living in developing countries. The Symposium addressed the challenges and opportunities for the effective implementation of these vaccines in the regions where they are most needed.

Currently, two vaccines are licensed for cholera, but are not yet readily used in the areas considered endemic for cholera, nor in children who are largely affected by the disease. Two recent studies have demonstrated that (a) cholera vaccine can be administered in mass immunization campaigns in areas where cholera is endemic with good protective effects and (b) that there appears to be good herd protection in vaccinated populations and non-vaccinated individuals. The remaining challenges include identifying and overcoming the obstacles to implementation of the vaccines in these settings. Similarly, the typhoid fever vaccines are licensed and have been proven to be effective in preventing the disease and can be administered in school-age populations with good protection given.

The present state of development of rotavirus vaccines indicates an urgent need to increase the global capacity to conduct multiple clinical trials in developing countries, particularly in Africa and Asia. These trials will need to evaluate safety, immunogenicity, and efficacy of the vaccine candidates in different populations and geographical areas which differ significantly in epidemiology of the virus and viral strain diversity and in the social, health, and economical background of the population—all of which may have a significant impact on final efficacy results.

The agenda of the Symposium (attached) included different sessions focused on different enteric vaccines, which allowed for discussion of priority issues which have been identified as major obstacles for the implementation of vaccines against cholera and typhoid fever and the introduction of rotavirus vaccines.

The participants of the Satellite Symposium were provided with an opportunity to attend all other sessions of the Forum to interact and share their experiences with experts working on other infectious diseases or areas. The linking of the Symposium with the Global Vaccine Research Forum has, thus, provided an ideal forum for cross-field interactions, exchange of information, and sharing of experiences between scientists against other priority infectious diseases, such as HIV, tuberculosis, malaria, and other infections, including enterotoxigenic *E. coli* and *Shigella*, with high-level participation from experts in the field of vaccines, including that from developing countries.

## Accomplishments and outcomes of the Symposium

The focus of the Symposium was typhoid fever and cholera (for which licensed vaccines have been available for a number of years), and rotavirus. The presentations addressed the question whether introduction plans for rotavirus, typhoid, and cholera vaccines are credible investment cases for developing countries.

The main conclusions of the Symposium were as follows:

- There is a global need for enteric vaccines, and rotavirus, typhoid, and cholera are all credible investment cases for developing countries; these investments should be based on the burden of disease, cost-effectiveness estimates, demonstration projects, and availability and financing of vaccines.
- As for rotavirus vaccines—for which an Accelerated Development and Introduction Plan (ADIP) already exists—a similar focused investment could be considered for typhoid vaccines.
- Good progress has been achieved with the rotavirus ADIP but it is very important to generate good rotavirus-efficacy data for Africa and Asia in addition to the existing data in Latin America, to assess the global performance of the new vaccines properly.
- Cholera vaccines need a double approach for high-endemic settings and control of epidemics. There is an urgent need for both recommendations on how to use the vaccine and on the question of the establishment of a stockpile.

For enteric vaccines in general, the following few hurdles should be overcome before wider use:

- More effective communication of the importance of enteric infections and the potential of vaccines to save lives in a cost-effective manner.
- Communication of the obvious—that vaccines are complements, and not replacements or threats, to other useful control methods.
- Advocacy that the search for the ‘ideal’ should not be the enemy of the ‘good’ and to use existing products while promoting improvements in existing vaccine formulations and research and development (R&D) into new vaccines.
- Definition of the complexities and provision of solutions to ‘challenges’, which are not ‘insurmountable problems’.

## More specifically, for typhoid fever vaccines

- Since antibiotic resistance is rising, and water and sanitation improvement is a long-term goal, vaccines are the only viable intervention to control typhoid in the short and medium terms.
- Typhoid fever causes significant mortality/morbidity and major direct and indirect economic losses; large-scale introduction of a typhoid vaccine is an opportunity to make a significant contribution to the Millennium Development Goals (MDGs) and to the objectives of GAVI.
- There are safe and effective, available, licensed vaccines; vaccination is feasible, acceptable, and affordable.
- There is a cost-competitive supply of vaccines, and there are ways to rationally vaccinate populations at highest risk in a financially-sustainable way.
- There is a perceived value and interest on the part of country-level decision-makers.

## More specifically, for rotavirus vaccines

- There is a need for safe, efficacious, and affordable vaccines. Rotavirus vaccines for the industrialized world will be available soon, but will a live, oral rotavirus vaccine work in infants in developing countries where it is needed most? This remains a key question.
- In addition to the two most advanced candidates, there are multiple additional vaccine candidates and producers interested in manufacturing rotavirus vaccines in developing countries.
- In view of the above, it is important that national regulatory authorities in developing countries continue to be strengthened by WHO and that work continues on standardization of quality control and safety (production process, facility, candidate strain…).

## More specifically, for cholera vaccines

- When should cholera vaccines be used?: (a) in high-endemic settings, and/or (b) as adjuncts for epidemic control? Guidance on this point is expected from WHO.
- A recently-published study in Mozambique showed that vaccine was highly effective against clinically significant cholera in an urban African population with a high prevalence of HIV infection.
- The establishment of a cholera vaccine stockpile has recently been discussed. However, there are still many challenges for the use of vaccines in disaster and outbreak situations.
- There is a need for WHO recommendations on how to use cholera vaccines.

Both Satellite Symposium and main Global Vaccine Research Forum were very well-attended with more than 50 and 150 participants respectively from all regions and representing all stakeholders of the fields of R&D of immunization and vaccine.

Extensive media coverage organized around both events allowed for effective advocacy and dissemination of key messages.

The report of the Symposium and Global Vaccine Research Forum has been completed and is being printed at WHO. It will be disseminated widely in the R&D of immunization and vaccine as hard copies and be posted on the Initiative for Vaccine Research website.

## Summary of the Symposium

The WHO Initiative for Vaccine Research and Department of Immunization, Vaccines and Biologicals plans to act on the conclusions and recommendations of the Symposium and notably in the following areas:

- In collaboration with GAVI partners, consider the opportunity for an investment case for typhoid vaccines.
- Collaborate with the WHO Global Cholera Task Force on the revision of recommendations on how to use the vaccines and on the establishment of a stockpile of potential vaccines.
- More effectively communicate the potential of vaccines against enteric infections.
- Advocate for the generation of credible rotavirus-efficacy data for Africa and Asia.
- Promote the development of more upstream rotavirus vaccine candidates by developing-country manu-facturers.
- Continue to strengthen national regulatory authorities in developing countries for the approval and monitoring of vaccine clinical trials and the registration of new vaccines.

**Table:** 

WORLD HEALTH ORGANIZATION			
INITIATIVE FOR VACCINE RESEARCH GLOBAL VACCINE RESEARCH FORUM			
12–15 June 2005, Salvador de Bahia, Brazil			
SATELLITE SYMPOSIUM ON THE CHALLENGES TO ENTERIC VACCINES			
AGENDA			
Moderator:	Jan Holmgren		
Rapporteur:	Duncan Steele		
08:00–09:00	Registration		
09:00–09:30	Global need for vaccines against enteric and diarrhoeal diseases in the developing world	—	Jan Holmgren
**Typhoid Fever Immunization—From Evidence Base to Implementation**		—	John Clemens
09:30–10:00	Control of typhoid fever in Pakistan through immunization	—	Anita Zaidi
10:00–10:30	The investment case of typhoid fever vaccination	—	Luis Jodar
10:30–10:45	Experiences with live oral typhoid vaccines	—	Mike Levine
10:45–11:00	Discussion		
11:00–11:20	Coffee break		
**Cholera Vaccines—A Public Health Tool?**		—	Ann-Mari Svennerholm
11:20–11:50	Cholera immunization—the Mozambique experience	—	Marcelino Lucas
11:50–12:20	When should cholera vaccines be used?	—	Claire-Lise Chaignat
12:20–12:50	Discussion		
12:50–14:00	**Lunch**		
**Rotavirus Vaccines—What Remains to be Done?**		—	Mike Levine
14:00–14:30	The hurdles and challenges ahead for rotavirus vaccines	—	Joe Bresee
14:30–14:45	Clinical trials for infants in developing countries	—	Duncan Steele
14:45–15.00	Challenges to oral delivery of vaccines	—	Martin Friede
15:00–15:30	Discussion		
15:30–16:30	Summary and wrap-up	—	Jan Holmgren

